# Functional disability and utilisation of long-term care in the older population in England: a dual trajectory analysis

**DOI:** 10.1007/s10433-022-00723-0

**Published:** 2022-08-02

**Authors:** Bo Hu, Javiera Cartagena-Farias, Nicola Brimblecombe

**Affiliations:** Care Policy and Evaluation Centre, Department of Health Policy, London School of Economics and Political Science, Clement’s Inn, London, WC2A 2AE UK

**Keywords:** Long-term care, Health inequality, Care inequality, Dual trajectory analysis, England

## Abstract

This study investigates the developmental trajectories of long-term care needs and utilisation in older people aged 65 years and over in England. The data came from the English Longitudinal Survey of Ageing (ELSA, waves 6–9, 2012–2018, *N* = 13,425). We conducted dual trajectory analyses to cluster people’s trajectories of care needs (measured by functional disability) and utilisation into distinct groups. We conducted logistic regression analyses to identify the factors associated with trajectory memberships. We identified three trajectories of long-term needs (low, medium, and high) and three trajectories of care utilisation (low, medium, and high). Both care needs and care hours increased with age, but the speed of increase varied by trajectory. Females, minority ethnic groups, people with low wealth, and those experiencing housing problems were more likely to follow the joint trajectories characterised by higher care needs and higher care intensity. People with low or medium care needs stayed in the same trajectories of care utilisation. In contrast, people in the high-needs trajectory followed divergent trajectories of care utilisation: 63% of them followed the trajectory of high care intensity and the rest (37%) followed the trajectory of medium care intensity. Lack of spouse care was the leading predictor of trajectory divergence (OR = 3.57, *p* < 0.001). Trajectories of care needs and utilisation are highly heterogeneous in later life, which indicates persistent inequalities over time. Single people with multiple functional limitations face an acute and enduring risk of inadequate care and unmet needs. The amount of support is as important as the availability of support.

**Supplementary Information:**

The online version contains supplementary material available at 10.1007/s10433-022-00723-0.

## Introduction

Preventing and delaying functional disability (e.g. difficulties in eating and housekeeping) in later life are the key to healthy ageing and can have a positive impact on older people’s quality of life. Functional disability is also one of the most important indicators of long-term care (LTC) needs (Geerts & Van den Bosch 2012). Long-term care provided by unpaid (informal) or formal caregivers plays a crucial role in modern welfare states because it helps older people to cope with functional declines, slows down the progression of care needs, and enables independence in life (Allen et al. 2014; Hu and Li 2020; Verbrugge & Jette 1994; World health Organisation 2015). The demand for long-term care is set to rise rapidly worldwide against the backdrop of global population ageing. Access to affordable long-term care for everyone is one of the key principles of the European Pillar of Social Rights set out by the European Commission (European Commission, 2017).

Turning the policy aspirations into reality is a challenge facing many governments which requires a systematic understanding of the patterns and determinants of functional capability and LTC utilisation. A number of studies have shown that the functional capability of older people differs markedly between demographic and socioeconomic groups, raising great concerns about health inequalities in the population (Basta et al. 2008; Caballero et al. 2017; Morciano et al. 2015). In parallel, prior research has investigated the distribution of LTC resources in the older population and reported that LTC utilisation is driven by care needs, demographic characteristics, socioeconomic status, and care availability (Katz et al. 2000; Larsson & Silverstein 2004; Suanet et al. 2012; Vlanchantoni et al. 2015).

Most of the existing studies have examined functional disability and LTC utilisation separately. Little is known about their joint development over time. Yet, this is a critical issue. It concerns how the inequalities of health and long-term care are interlinked or reinforce each other as time goes by, which may lead to cumulative disadvantages or multiple adverse outcomes in life. Moreover, it touches upon the fundamental issue of horizontal equity. Care equity, namely equal care for equal needs, is the inherent logic of welfare states where the stated aim of care provision is to promote social justice and distributive fairness in society (Carrieri et al. 2017; Department of health and Social Care, 2021; European Commission 2017; Floridi et al. 2021; HM Government 2014; Rodrigues et al. 2018). Although an overwhelming amount of evidence has demonstrated the strong correlation between functional disability and care utilisation, this does not necessarily mean that care provision always meets care needs for different groups of people. An investigation into the dual trajectories will clarify whether or not and to what extent the developmental trajectory of care utilisation deviates from that of functional disability at different time points in life. Care inequity in the older population is an outcome as much as a process.

The existing research on the trajectories of functional disability has found considerable heterogeneity of disability progression in later life and contributed to the understanding of the ageing experience and health inequality from a life course perspective (Fors et al. 2022; haviland et al. 2011; Li & Zhang 2018; Zimmer et al. 2012). Females are found to follow heightened disability trajectories than males in later life (Li & Zhang 2018; Zimmer et al. 2012). Gender-based differences are especially pronounced in Eastern and Southern Europe rather than in Northern and Western Europe (Fors et al. 2022). Meanwhile, people with high socioeconomic status (e.g. high educational attainment) often follow the trajectories of high functional capability (Li & Zhang 2018).

Trajectory analyses of LTC utilisation provide useful information about the duration of care inequality in the population, reveal the dynamics between different sources of care, and inform policy reforms that may be ‘life-changing’ to care recipients (Geerts & Van den Bosch 2012; Hu 2020; Penning et al. 2018). Li (2005) found that older people received markedly less informal care in the 2 years after they had access to publicly funded home care. Drawing on data collected from 473 community-dwelling older individuals over 15 years in Denmark, Kjær and Siren (2020) identified four clusters of care trajectories and found that whether individuals used formal or informal care in the long term was associated with the levels of care needs and the availability of social support networks.

Building upon the existing literature, this study investigates the dual trajectories of functional disability and care utilisation in community-dwelling older people in England. We use functional disability to measure long-term care needs and examine the hours of long-term care that older people receive (i.e. care intensity). Many studies have focussed on whether or not people receive care, but evidence on the hours of care is relatively sparse. Drawing on longitudinal data collected in a national survey, we conduct latent trajectory analysis to cluster the diverse trajectories into distinct groups. The study aims to answer three research questions: (1) What are the levels of inequalities in care needs and utilisation in the older population, and how do they vary over time and according to people’s demographic and socioeconomic status? (2) For people following the same trajectories of care needs, do their trajectories of care intensity diverge? (3) What are the drivers of divergence in trajectories?

## Research methods

### Data

**Fig. 1:**
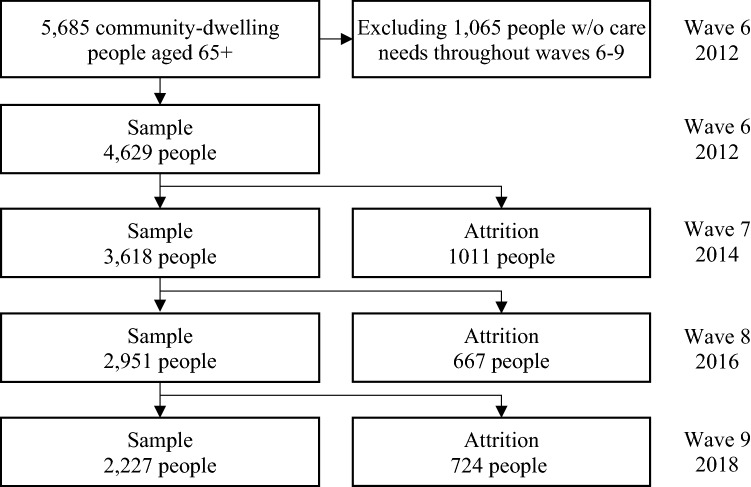
Sample structure, ELSA, waves 6–9 Attrition sample includes people who died or were lost to follow-up

The data in this study came from the English Longitudinal Survey of Ageing (ELSA), which collected health and ageing-related information from a nationally representative sample of people aged 50 and over in England. The baseline survey took place in 2002, with follow-up surveys conducted every 2 years (NatCen Social Research, 2020). Our study drew on data in waves 6–9 (2012–2018), which contained detailed information on long-term care utilisation that was not available in previous waves. We excluded 1,056 people without care needs throughout the four waves and focused on the 4,629 community-dwelling older people who were aged 65 years and over in wave 6 and developed care needs in at least one wave. The sample size across the four waves is 13,425 (Fig. 1).

### Measurements

Long-term care needs were measured by people’s ability to perform daily tasks. The ELSA asked participants to report whether or not they had difficulties in performing six activities of daily living (ADLs; dressing, walking across the room, bathing, eating, getting in and out of bed, and using the toilet), four instrumental activities of daily living (IADLs; shopping, taking medications, working around the house, and managing money), and three mobility tasks (walking 100 yards, climbing several flights of stairs without rest, and climbing one flight of stairs without rest). To ensure that these questions related to long-term care needs, the survey emphasised that participants should only answer affirmatively if the issues arose because of physical, mental, emotional, or memory problems and were expected to last more than three months. We created a variable to measure long-term care needs by adding up the number of difficulties participants reported in performing the 13 tasks. A higher number of difficulties indicates a higher level of care needs. This follows previous studies that have shown that there is a single latent dimension underlying the functional capability to perform ADL, IADL and mobility tasks (Kingston et al. 2012; Spector & Fleishman 1998). The Cronbach alpha of the 13 items ranged from 0.847 to 0.862 across the four waves in our sample. For each task, survey participants were further asked whether they received help from informal (unpaid) or formal caregivers and, if so, how many hours of care they received from each caregiver in a week. We created a care intensity variable by adding up the hours of care received. More hours of care indicate a higher level of care intensity. We investigated the combined total hours of care. We also examined the hours of informal care and formal care, respectively.

The Behavioural Model of Care Utilisation was used to guide the investigation into factors that may predict the co-development of care needs and utilisation (Andersen & Newman 2005). We analysed five demographic variables including age, gender, marital status, whether or not living with children, and ethnicity. Marital status was a binary variable: 0 = never married, widowed, separated, or divorced and 1 = married. The ethnicity variable was dichotomised: 0 = white ethnicity and 1 = ethnic minority. We focused on four socioeconomic factors: equivalised weekly income, total non-pension wealth, educational qualifications, and occupation. Both income and wealth were continuous variables. The education variable had three categories: no formal education, primary/secondary education, and higher education or equivalent qualifications. The occupation variable had three categories: managerial/professional, intermediate, and routine/manual occupations. Two environmental factors, housing problems and geographical location, were included in the analysis. The ELSA asked participants whether their housing had any of the following issues: a shortage of space, noise from neighbours, street noise, insufficient light, street pollution, dampness, water leaks, bad condensation, plumbing problems, rot/decay, insects/rats, and a low temperature in winter. We created a variable by counting the total number of housing issues. We combined the nine English regions into three categories: Northern England, the Midlands, and Southern England.

### Statistical analysis

Our analysis consisted of two steps. In the first step, we built a dual trajectory model to classify people into groups based on their joint memberships in trajectories of care needs and utilisation (Stata syntax: *traj*). Following Nagin (2005), the likelihood function of our dual trajectory model was specified as follows:

1 $$P\left({\varvec{N}}, {\varvec{C}}\right)=\sum_{j}\sum_{k}{\pi }_{jk}\times {f}^{j}({\varvec{N}})\times {f}^{k}\left({\varvec{C}}\right)$$

where ${\varvec{N}}$ and ${\varvec{C}}$ denote an older adult’s sequences of care needs and care intensity (namely total combined hours of unpaid and formal care), respectively, over the four waves of the ELSA survey. ${\pi }_{jk}$ denotes the joint probability of membership in the (latent) trajectory j for care needs and trajectory k for care intensity. ${f}^{j}(N)$ and ${f}^{k}(C)$ are the multivariate probability density functions for the sequences of care needs and intensity, respectively. Following Nagin (2005), three assumptions were made in these probability density functions. First, we assumed that attrition and memberships of latent trajectories were independent (i.e. random attrition assumption). Second, both care needs and intensity were assumed to be sequentially independent conditional on belonging to a specific trajectory (i.e. conditional independence assumption):

2 $${f}^{j}\left({\varvec{N}}\right)=\boldsymbol{ }\prod_{t}{f}^{j}\left({N}_{t}\right)$$

3 $${f}^{j}\left({\varvec{C}}\right)=\boldsymbol{ }\prod_{t}{f}^{j}\left({C}_{t}\right)$$

where $t$ denotes a specific wave of the ELSA survey ($t$=1–4). Finally, conditional on their respective trajectories, both care needs and intensity were assumed to have a censored normal distribution linked to a polynomial function of the age variable of the following form:

4 $${N}_{t}^{*}={\beta }_{0}^{j}+{\beta }_{1}^{j}\times {Age}_{t}+{\beta }_{1}^{j}\times {Age}_{t}^{2}+{\varepsilon }_{t}$$

5 $${C}_{t}^{*}={\beta }_{0}^{k}+{\beta }_{1}^{k}\times {Age}_{t}+{\beta }_{2}^{k}\times {Age}_{t}^{2}+{\varepsilon }_{t}$$

where ${N}_{t}={N}_{t}^{*}$ if $0\le {N}_{t}^{*}\le 13$, ${N}_{t}=0$ if ${N}_{t}^{*}<0$, and ${N}_{t}=13$ if ${N}_{t}^{*}>13$. ${{C}_{t}=C}_{t}^{*}$ if $0\le {C}_{t}^{*}\le 168$, ${C}_{t}=0$ if ${C}_{t}^{*}<0$, and ${C}_{t}=168$ if ${C}_{t}^{*}>168$. Some older people may not receive any care, while others may receive around-the-clock care that amounts to 168 h per week at the maximum. Following the previous studies, we scaled the age variable to one-tenth of its original value to facilitate the search for the value that maximises the likelihood function (Nagin 2005; Zimmer et al. 2012).

The AIC and BIC were used to select the model with the optimal number of groups. They were calculated as follows:

6 $$BIC=\mathit{log}\left(L\right)-0.5\times k\times log(N)$$

7 $$AIC=log\left(L\right)-0.5\times k$$

where $L$ is the value of the maximised likelihood of the model, $k$ is the number of the parameters, and $N$ is the sample size. A larger AIC or BIC value indicates a better model. We calculated the average posterior probability (APP) and the odds of correct classification (OCC) to assess the model adequacy. APP should be at least 0.7 and OCC at least 5 for all groups (Keeney et al. 2019; Nagin 2005). Based on the analysis in this step, we created categorical variables, which were fed into the analyses in the next step. Following the previous study (Zimmer et al. 2012), we plotted the predicted care needs and care utilisation against chronological age for each trajectory, which allowed us to better visualise the speed of change. Such an analysis assumed that the birth cohorts did not have a major impact on the shape of a trajectory.

In the second step, we conducted logistic regression analyses to investigate the factors associated with the joint memberships. The outcomes were the trajectory variables created in the first step. Regarding the predictors, we focused on the aforementioned demographic, socioeconomic, and environmental factors reported in wave 6 of the survey. The proportions of missing values in the predictors were generally low (see Supplementary material). The multiple imputation with chained equations (MICE) technique was used to sequentially impute all of the predictors with missing values (20 imputed datasets of predictors). Stata version 16 was used for analyses.

## Results

**Fig. 2:**
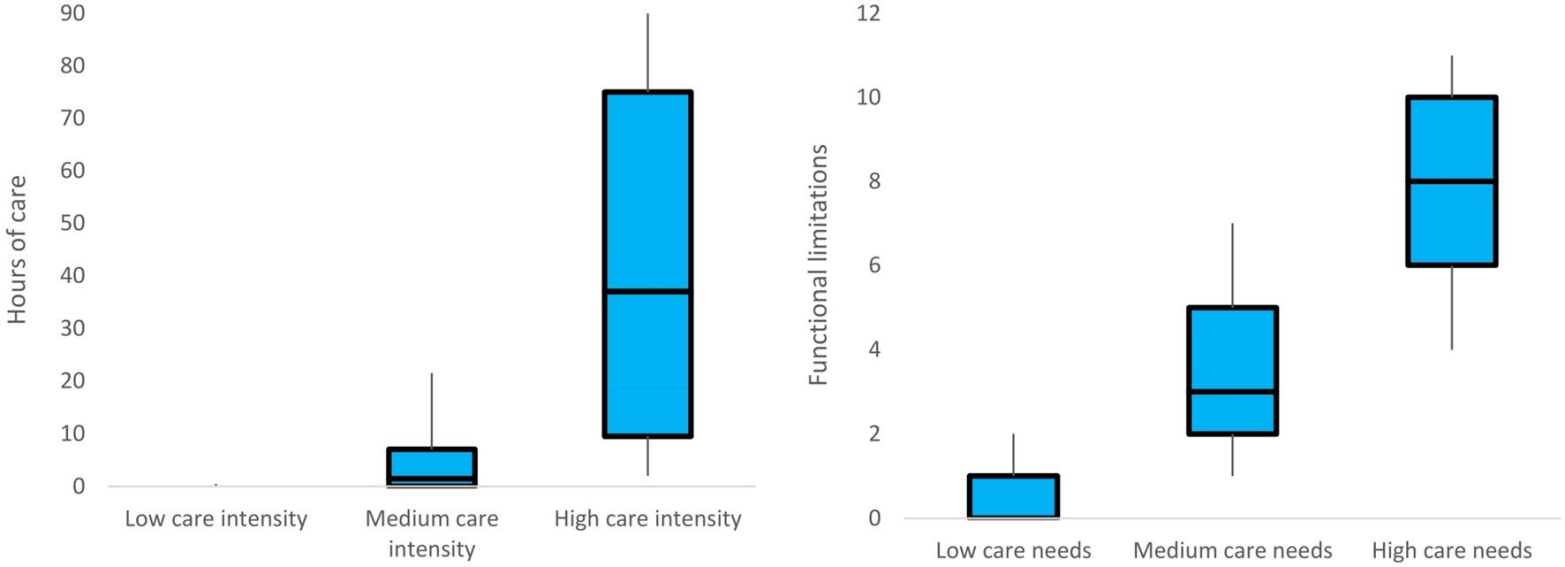
Boxplots of care intensity and functional limitations according to trajectory memberships (*N* = 13,425) Left panel: sample distribution of the hours of long-term care according to the trajectories of care intensity; Right panel: sample distribution of the number of functional limitations according to the trajectories of care needs; The box covers the interquartile range; The horizontal line splitting the box is the median; The two whiskers extend to the 10th percentile and 90th percentile of the distribution, respectively

Our analysis shows that the optimal model is the one with three trajectories of long-term care needs and three trajectories of care utilisation, respectively (Table 1). This model reported higher AIC and BIC scores than those with fewer trajectories. The models with four or more trajectories failed to converge. For each trajectory, the APP was above the threshold of 0.7, and the OCC was above the threshold of 5, which provided a strong indication that our dual trajectory model was adequate. The boxplots in Fig. 2 show that the model has a high degree of latent trajectory separation. This implies a high degree of homogeneity within each group (Collins & Lanza 2010, p. 64). Notably, there is no overlap in interquartile ranges between two adjacent trajectories (i.e. low vs. medium trajectories or medium vs. high trajectories) and no overlap in whiskers between two extreme trajectories. This confirms that our model has a good fit.

**Table 1:** Results of dual trajectory modelling (*N* = 13,425)

	Trajectories of care needs			Trajectories of care intensity		
	Group 1 (Low)	Group 2 (Medium)	Group 3 (High)	Group 1 (Low)	Group 2 (Medium)	Group 3 (High)
*Coefficient (standard error)*						
Intercept	9.94*	37.93***	4.45	144.61	64.05	−41.23
	(4.40)	(5.94)	(11.18)	(96.27)	(71.83)	(174.12)
Linear term of age variable	−3.82***	−10.88***	−1.06	−70.33***	−35.04	−6.22
	(1.14)	(1.54)	(2.93)	(24.31)	(18.4)	(45.86)
Quadratic term of age variable	0.32***	0.82***	0.19	5.95	3.36***	2.40
	(0.07)	(0.10)	(0.19)	(1.53)***	(1.17)	(3.00)
*Number of observations*						
Wave 6	2,820	1,367	442	2,819	1,497	313
Wave 7	2,189	1,120	309	2,189	1,208	221
Wave 8	1,792	928	231	1,792	993	166
Wave 9	1,382	694	151	1,382	737	108
Waves 6–9	8,183	4,109	1,133	8,182	4,435	808
*Post-estimation diagnostics*						
APP	0.92	0.88	0.90	0.92	0.87	0.90
OCC	7.6	14.3	113.2	7.6	14.5	82.3
BIC	−47,052					
AIC	−46,937					

^*^p < 0.05, **p < 0.01, ***p < 0.001; APP: average posterior probability; OCC: odds of correct classification

**Fig. 3:**
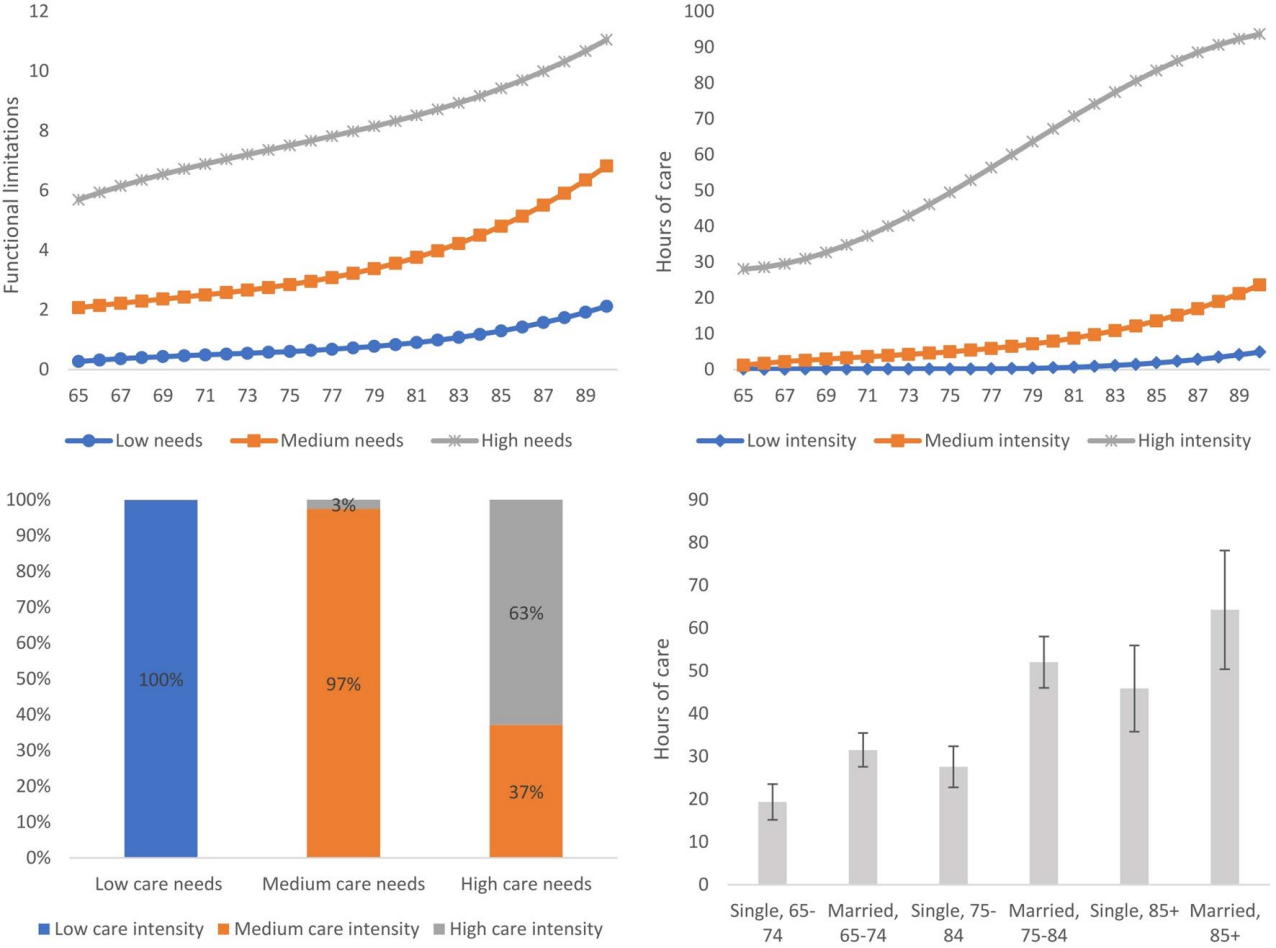
Trajectory memberships of long-term care needs and intensity (*N* = 13,425) Upper left panel: Trajectories of predicted care needs (measured by the number of functional limitations) according to age; Upper right panel: Trajectories of predicted care intensity (measured by weekly h of unpaid and formal care) according to age; lower left panel: proportion of people in different trajectories of care intensity conditional on trajectories of care needs; lower right panel: average h of care according to marital status and age in older people following the high-needs trajectory (95% confidence interval plotted on the mean)

Our model classified people into low, medium, and high trajectories of LTC needs. Among the 4,629 people in wave 6, 2,820 people followed the low-needs trajectory (Table 1). Due to attrition, 1,382 of them remained in this trajectory by wave 9. There were 8,183 observations in this group across four waves. In this trajectory, the predicted number of functional difficulties increased from 0.3 at the age of 65 years old to 2.1 at the age of 90 and over (upper left panel in Fig. 3). Among the 4,629 people in wave 6, a total of 1,367 people were in the trajectory of medium care needs. There were 4,109 observations in this group across the four waves (Table 1). The predicted number of functional difficulties increased from 2.1 at the age of 65 years old to 6.8 at the age of 90 and over. Notably, the rise in care needs accelerated after the age of 70. A total of 1,133 observations were classified into the trajectory of high care needs (Table 1). The predicted number of functional difficulties increased from 5.7 at the age of 65 years old to 11.0 at the age of 90 and over. In this trajectory, the rise in care needs started to accelerate at around age 75. The attrition rate in the low-needs trajectory was very close to that in the medium-needs trajectory but lower than that in the high-needs trajectory (Table S3 in Supplementary Materials). Overall, the violation of the random attrition assumption was rather limited.

**Fig. 4:**
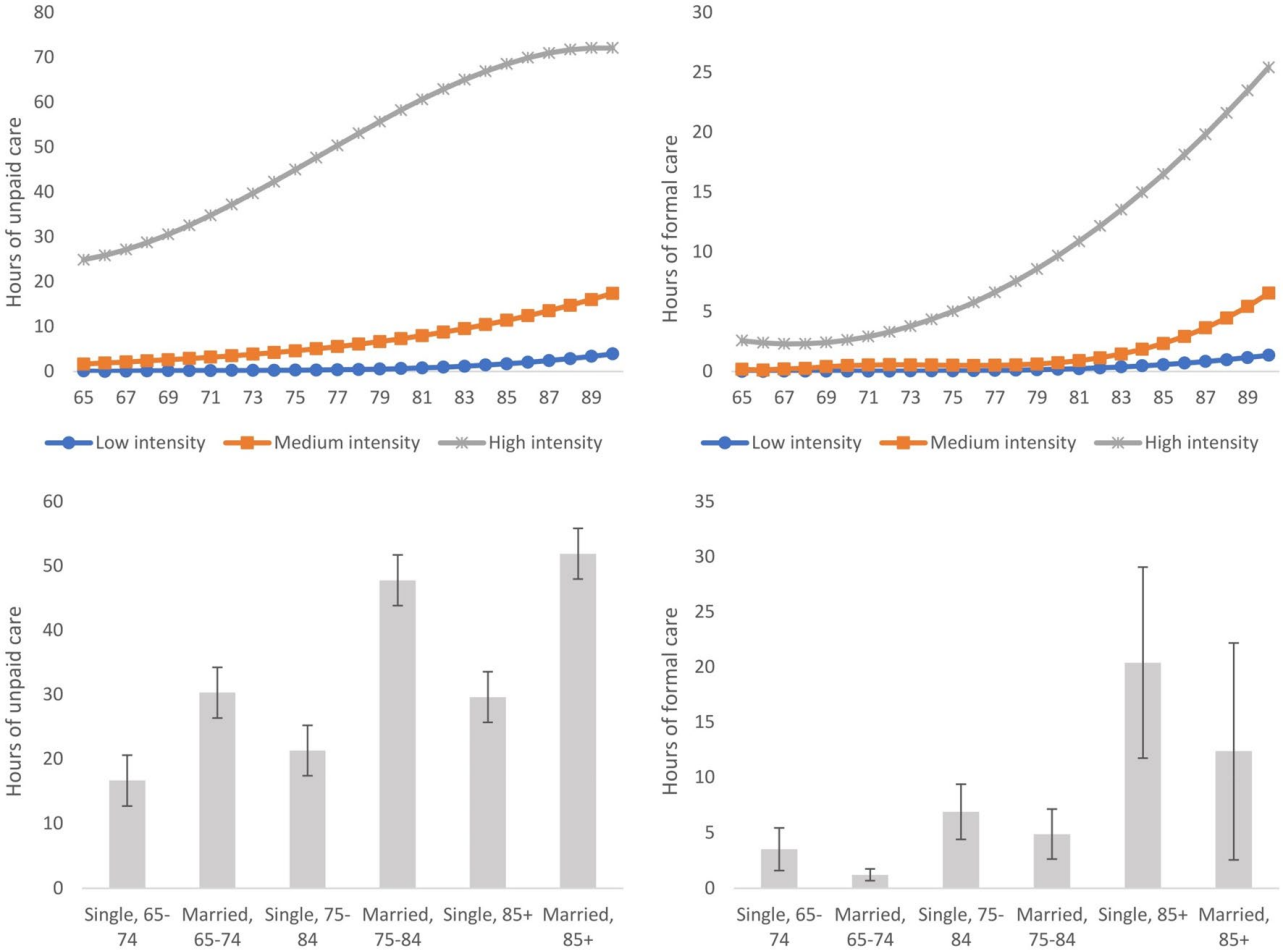
Trajectory memberships of long-term care intensity according to sources of care (*N* = 13,425) Upper left panel: Trajectories of predicted informal care intensity according to age; Upper right panel: Trajectories of predicted formal care intensity according to age; lower left panel: average h of informal care according to marital status and age in older people following the high-needs trajectory (95% CI plotted on the mean); lower right panel: average h of formal care according to marital status and age in older people following the high-needs trajectory (95% CI plotted on the mean)

Our model classified people into low, medium, and high trajectories in terms of long-term care utilisation. A total of 8,182 observations were classified into the trajectory of low care intensity across the four waves. The utilisation of care was predicted to increase from 0.1 h per week at the age of 65 to 4.9 h per week at the age of 90 and over (upper right panel in Fig. 3). A total of 4,435 observations were classified into the medium-intensity trajectory. Care utilisation was predicted to rise from 1.2 h per week at the age of 65 to 23.6 h per week at the age of 90 and over. For the 808 observations in the high-intensity trajectory, care utilisation was predicted to increase from 28 h per week at the age of 65 to 93.7 h per week at the age of 90 and over. We further broke down each trajectory of care utilisation into trajectories of unpaid and formal care (upper left and right panel in Fig. 4). It can be noted that for people in the high-intensity trajectory, the increase in the hours of informal care started to slow down at around the age of 80, whereas the increase in the hours of formal care accelerated after the age of 70. The random attrition assumption was plausible for the low-intensity and medium-intensity trajectories, but was mildly violated for the high-intensity trajectory (Table S4 in Supplementary Materials).

The lower left panel of Fig. 3 shows the joint memberships of older people. The low-need and low-intensity trajectories were completely overlapped. All of the people classified into the low-needs trajectory were in the low-intensity trajectory, and none of them followed the medium- or high-intensity trajectory. However, for people with a higher level of care needs, their trajectories of care intensity became divergent. Conditional on belonging to the medium-needs trajectory, 97% of people followed the medium-intensity trajectory and 3% followed the high-intensity trajectory. None of them followed the low-intensity trajectory. Among the people in the high-needs trajectory, 37% followed the medium-intensity trajectory and 63% followed the high-intensity trajectory. In total, our dual trajectory model identified five joint memberships in trajectories: low-needs/low-intensity, medium-needs/medium-intensity, medium-needs/high-intensity, high-needs/medium-intensity, and high-needs/high-intensity trajectories (see Table S1 in Supplementary Material for more details).

Table 2 reports the characteristics of the sample in wave 6 of the survey (*n* = 4,629). The average age of our sample was 75.0 years old. Females accounted for 56% of the sample, and 2.3% were in the minority ethnic groups. In addition, 60% of the older people were married, and 11% were living with adult children in the same household. One-quarter of the sample received secondary education or had equivalent qualifications, and another quarter received higher education. The average wealth amounted to £0.3 million. People in our sample on average reported 0.4 housing problems. Three-quarters of them experienced no housing problems, and 9% reported two or more housing problems.

**Table 2:** Sample characteristics in wave 6

	Proportion (%) or mean	n
*Demographic factors*		
Age (years old)	75.0	4,629
Gender		
Male	43.7	2,022
Female	56.3	2,607
Ethnicity		
White	97.7	4,522
Ethnic minority	2.3	106
*Marital status*		
Single	40.2	1,863
Married	59.8	2,765
Living with children		
No	89.3	4,132
Yes	10.7	497
*Socioeconomic factors*		
Education		
No qualification	48.5	2,208
Secondary education	26.2	1,189
Higher education	25.3	1,153
Most recent occupation		
Managerial/professional occupation	29.3	1,354
Intermediate occupation	25.3	1,171
Routine/Manual/other occupation	45.4	2,104
Wealth (£mil)	0.3	4,518
Income (£thousand per week)	0.3	4,518
*Environmental factors*		
Average number of housing problems	0.4	4,629
No housing problems	74.1	3,432
One housing problem	16.8	779
Two or more housing problems	8.9	418
Geographical locations		
Northern England	28.4	1,313
The Midlands	22.2	1,026
Southern England	49.4	2,284

Table 3 shows the association between the predictors in wave 6 and the joint memberships in trajectories. The number of people in the joint trajectories characterised by medium care needs and high care intensity was tiny (*n* = 35), so people in this group were not included in the regression analyses. As demonstrated in column 2, females were more likely than males to be in the medium-needs/medium-intensity trajectories rather than in the low-needs/low-intensity trajectories. Minority ethnic people were more likely than white people to follow the medium-needs trajectory rather than the low-needs trajectory. Socioeconomic status matters. People who had a higher level of education, a managerial/professional job as their most recent occupation, and a higher level of wealth were less likely than people with a lower socioeconomic status to follow the medium-needs trajectory as opposed to the low-needs trajectory. The correlation coefficient between the wealth and income variables was 0.49 (*p* < 0.001). Including both variables may introduce a certain degree of multicollinearity into the models. In addition, the income variable was not statistically significant regardless of whether the wealth variable was included. Therefore, we decided not to include the income variable in the final model. It is also worth noting that people experiencing multiple housing problems were more likely than those in better housing conditions to follow the medium-needs trajectory as opposed to the low-needs trajectory (Column 2, Table 3).

**Table 3:** Factors associated with joint trajectories: binary logistic regression analyses

	Low needs & low intensity vs. Medium needs & medium intensity	High needs & medium intensity vs. high needs & high intensity
	Odds ratio (standard error)	Odds ratio (standard error)
*Female*	1.35***(0.1)	1.30 (0.29)
Ethnic minority	2.02**(0.47)	1.58 (0.92)
Married	0.83*(0.06)	3.57***(0.84)
Living with children	1.05 (0.12)	1.75 (0.56)
Secondary education	0.93 (0.08)	1.56 (0.45)
Higher education	0.80*(0.08)	1.25 (0.43)
Managerial/Professional	0.87 (0.09)	0.66 (0.22)
Intermediate job	1.31**(0.12)	1.29 (0.4)
Wealth	0.76* (0.08)	2.40 (1.52)
Housing problems	1.71***(0.05)	1.04 (0.12)
Midlands	1.03 (0.1)	0.61 (0.18)
Southern England	0.82*(0.07)	0.39**(0.11)

^*^p < 0.05, **p < 0.01, ***p < 0.001

For people following the high-needs trajectory, marital status was the most important predictor of care intensity, and none of the other factors was statistically significant (column 3). The odds of belonging to the high-needs/high-intensity trajectories (as opposed to the high-needs/medium-intensity trajectories) were more than three times larger in married people than in single people. Such a difference can be visualised in the lower right panel in Fig. 3. Conditional on belonging to the high-needs trajectory, married people received significantly more hours of care than single people, regardless of their age. Again, we broke down the total hours of long-term care into hours of formal and informal care (lower left and right panels in Fig. 4). It can be noted that people utilised different sources of care to meet their needs: single people received more hours of formal care and fewer hours of informal care than married people.

## Discussion

This study investigated the simultaneous trajectories of functional disability and utilisation of long-term care in older age in England. Drawing on longitudinal data from national surveys, our study made several novel contributions to the literature. First, the latent trajectory analysis provided rich information about the inequality in the older population. We found that people followed three distinct trajectories of care needs and care utilisation, respectively. These inequalities were highly persistent throughout later life, and there is little sign that they were diminished over time. Previous research has shown that inequality in care needs is strongly driven by socioeconomic status and ethnicity (Basta et al. 2008; Daoud et al. 2009; Evandrou et al. 2016; Hu et al. 2022; McMunn et al. 2009). Our study adds that those factors also influence inequalities in trajectories of functional disability, which in turn leads to unequal trajectories in care utilisation.

Second, both care needs and care intensity rise with age, but the speed of increase varies across different trajectories. For older people following the trajectory characterised by high care intensity, there is a notable slowdown in the increase in informal care intensity after the age of 80. In comparison, the increase in formal care intensity accelerated after the age of 80, regardless of care trajectory. The proportion of people aged 80 and over in the older population is projected to keep rising in the coming decades (Office for National Statistics 2019). Our findings imply that, due to the lack of potential carers in this group of people, the increase in the utilisation of formal care is likely to outpace that of informal care. It is important that the long-term care system is prepared for this structural shift in care utilisation. In addition, it would be useful for the government to take preventative action to slow down the increases in care needs and utilisation. Improving the housing conditions of older people may play a constructive role, given the strong association between housing problems and trajectories of care needs identified in our study.

Third, care inequity varies by the trajectory of care needs. For older people with a lower level of care needs, nearly all of them followed the same trajectories of care utilisation. however, divergence emerged in older people following the high-needs trajectory. The international literature has reported consistent evidence from different countries that long-term care needs are strongly correlated with care utilisation (Larsson et al. 2006; McAuley et al. 2009; Vlanchantoni et al. 2015). In comparison, our study shows that care needs and utilisation are not always aligned with each other when we classify people into distinct groups. It seems that, for some people, the amount of care they receive might not keep up with their rising needs and it is increasingly difficult to fully meet the needs of older people with severe functional limitations. Put differently, the risk of unmet needs increases with the level of needs.

The regression analysis shows that the lack of access to spouse care is the leading reason for receiving fewer hours of care. Drawing on data from nine European countries, Geerts and Van den Bosch (2012) reported that older people transitioned to formal care once informal care became unavailable. Our study shows that such a relationship between formal care and informal care can be observed in terms of care hours as well: single people tended to receive more hours of formal care and fewer hours of informal care. Most importantly, we have found that, for people with severe care needs, formal care did not entirely offset the loss of spouse care, which led to divergent care trajectories. People in this group might have access to long-term care from other sources after the loss of informal care, but the overall level of care was markedly reduced. These findings suggest that the government may want to vamp up support for single older people experiencing multiple functional limitations even though they have already been receiving care. The central issue here concerns not only the availability of care and support but also the adequacy of support in meeting older people’s care needs.

Despite its strengths and novel contributions, the limitations of the study should also be duly noted. First, following Nagin (2005), we made the random attrition assumption in our dual trajectory modelling. Post-estimation analyses showed that this was a plausible assumption in the trajectories characterised by low or medium care needs as well as low or medium care intensity. The attrition rates were higher in the high-needs and high-intensity trajectories. Since the violation of this assumption is relatively mild, it seems unlikely that our results in relation to the classification and the regression analyses were seriously biased. We also tried to relax this assumption by parameterising non-random attrition across the trajectories, but the dual trajectory models became too complicated to converge. Second, this study adopted the synthetic cohort approach to examine how care needs and intensity changed with age. Like other studies using this approach, the cohort effects were not accounted for (Zimmer et al. 2012). Fors et al. (2022) investigated the trajectories of functional disability in older people in Europe born in different cohorts. They did not find strong evidence of cohort-specific differences. Therefore, it seems reasonable to assume that parameterising cohort effects in our study will not lead to notable changes in the trajectories of functional disability. Once more waves of ELSA data are available, future research could benefit from the analyses of the cohort-specific trajectories of long-term care utilisation. So far, there has been little evidence on this issue.

## Conclusion

The dual trajectory analysis provides useful information about the dynamics of, and the interaction between inequalities of care needs and care utilisation in the older population in England. Those inequalities are an outcome as much as a process, and preventative measures and interventions are needed to tackle the persistent inequalities throughout later life. Single people with complex long-term care needs face an acute and enduring risk of unmet care needs, which calls for heightened support for this particular group of people. The amount of support is as important as the availability of support.

## Supplementary Information

Below is the link to the electronic supplementary material.

Supplementary file1 (DOCX 14 KB)

## Data Availability

The ELSA data used in this study are publicly available in the UK Data Service repository. The Stata code that can reproduce the results reported in this study will be available upon request.
