# Supplementary material for: Functional disability and utilisation of long-term care in the older population in England: a dual trajectory analysis
Source: Eur J Ageing. 2022 Aug 2;19(4):1363–73. doi: 10.1007/s10433-022-00723-0 (PMC9729679; doi:10.1007/s10433-022-00723-0)
Supplement: Supplementary file 1 — Supplementary file1 (DOCX 14 KB) [file 10433_2022_723_MOESM1_ESM.docx]

**Supplementary material**

***Memberships of joint trajectories***

Our model identified three trajectories of care needs and three trajectories of care utilisation. In theory, there are nine joint group memberships. In reality, there are only five joint groups because some groups have no observations. This is shown in table A1. Among the 13,425 people across four waves of the survey, 8,182 people were classified into the joint trajectory of low care needs and low care intensity, 4,011 people followed the trajectory of medium care needs and medium care intensity, 423 people were in the trajectory of high care needs and medium care intensity, and 710 people were classified into the trajectory of high care needs and high care intensity.

Table S1 Number of people in joint groups (waves 6-9, ELSA, N=13,425)

|  | Low care intensity | Medium care intensity | High care intensity | Total |
| --- | --- | --- | --- | --- |
| Low care needs | 8,182 | 1 | 0 | 8,183 |
| Medium care needs | 0 | 4,011 | 98 | 4,109 |
| High care needs | 0 | 423 | 710 | 1,133 |
| Total | 8,182 | 4,435 | 808 | 13,425 |

Table S2 Proportion of missing values in predictors of trajectories (N=4,629)

|  | Proportion of missing values (%) |
| --- | --- |
| Age | 0 |
| Gender | 0 |
| Ethnicity | <0.1 |
| Marital status | <0.1 |
| Living with children | 0 |
| Education | 1.7 |
| Occupation | 0 |
| Income | 2.4 |
| Wealth | 2.4 |
| Housing problems | 2.7 |
| Region | 0.13 |

Table S3 Proportion of people in the attrition sample according to memberships of care needs

| Attrition | Low care needs | Medium care needs | High care needs | Total |
| --- | --- | --- | --- | --- |
| No | 80.8% | 82.6% | 72.7% | 80.6% |
| Yes | 19.2% | 17.5% | 27.3% | 19.4% |
| n | 8,183 | 4,109 | 1,133 | 13,425 |

Table S4 Proportion of people in the attrition sample according to memberships of care utilisation

| Attrition | Low care intensity | Medium care intensity | High care intensity | Total |  |
| --- | --- | --- | --- | --- | --- |
| No | 80.8% | 81.8% | 72.9% | 80.6% |  |
| Yes | 19.2% | 18.2% | 27.1% | 19.4% |  |
| n | 8,182 | 4,435 | 808 | 13,425 |  |
